# Multiple distant metastases arising from a single, low-grade rectal neuroendocrine tumor: an autopsy case report

**DOI:** 10.1186/s13256-023-03854-9

**Published:** 2023-03-27

**Authors:** Keng Wee Goh, Hiroshi Yoshida, Ichiro Miura, Chisako Miura, Kazuaki Norita, Takayuki Ii, Hideaki Yamanami, Koju Kobayashi

**Affiliations:** 1 Junior Residency, Obihiro Daiichi Hospital, Obihiro, Japan; 2 Department of Palliative Care, Obihiro Daiichi Hospital, Obihiro, Japan; 3 Department of Gastroenterology Medicine, Obihiro Daiichi Hospital, Obihiro, Japan; 4 Department of Surgery, Obihiro Daiichi Hospital, Obihiro, Japan; 5 Department of Clinical Laboratory, Hokkaido Institutional Society Obihiro Hospital, Obihiro, Japan; 6 Department of Diagnostic Pathology, Hokkaido Institutional Society Obihiro Hospital, Obihiro, Japan

**Keywords:** Autopsy, Case report, Neoplasm metastasis, Neuroendocrine tumors, Rectum

## Abstract

**Background:**

Rectal neuroendocrine neoplasms are rare epithelial neoplasms of the rectum. The incidence of these tumors has increased over the past decades. However, many questions remain unanswered regarding their clinicopathology, including the possible mechanisms in which these tumors may grow and metastasize.

**Case presentation:**

In this case report, we report the findings of an autopsy of a 65-year-old Japanese woman diagnosed with multiple liver metastases from a single, low-grade rectal neuroendocrine tumor. The diagnosis was made in late 2018 to early 2019, and subsequently the patient underwent several rounds of standard chemotherapy. However, due to unfavorable side effects, she opted for palliative care at our hospital instead from December 2020. The patient’s condition was generally stable for the next 17 months, but in May 2022, she was hospitalized for increased abdominal pain. Despite enhanced pain control therapy, she eventually passed away. An autopsy was conducted to determine the exact cause of death. The primary rectal tumor was found to be small, but showed strong histological evidence of venous invasion. Metastases in the liver, pancreas, thyroid gland, adrenal glands, and vertebrae were also present. On the basis of the histological evidence obtained, we deduced that the tumor cells may have mutated and gained multiclonality as they spread vascularly to the liver, contributing to the distant metastases.

**Conclusions:**

The results from this autopsy may provide an explanation for the possible mechanism by which small, low-grade rectal neuroendocrine tumors metastasize.

## Introduction

Neuroendocrine neoplasms (NENs) are rare epithelial neoplasms mostly occurring in the hypothalamus, parathyroid glands, lungs, pancreas, and gastrointestinal tract [1]. Within the gastrointestinal tract, rectal NENs are the most common in Japan [2], with an incidence rate of 4.52 per 100,000 population [3, 4]. The incidence of rectal NENs have increased during the past two decades [4, 5], and reasons for such trends are suggested to be increased participation in colonoscopy screenings, as well as improved colonoscopy techniques [6].

Rectal NENs are classified on the basis of biopsy findings [7]. Well-differentiated neoplasms with lower Ki-67 levels are classified as neuroendocrine tumors (NETs) grades (G) 1–3, with a higher grade representing a lower level of differentiation and a higher Ki-67 index. Meanwhile, poorly differentiated neoplasms are classified as neuroendocrine carcinomas (NECs). G3 NETs and NECs are more aggressive than NETs of lower grades, with a higher risk of distant metastasis [8].

Tumor size is another factor that affects the risk of distant metastasis. One retrospective study reported a metastatic risk of 60–80% for primary tumors larger than 20 mm [9]. Depth of invasion, the presence of regional lymph node metastases, as well as atypical histology have also been reported as risk factors for distant metastasis [10], although evidence has been elusive.

Most rectal NENs are asymptomatic [11], and are mostly diagnosed incidentally during endoscopic procedures for colorectal cancer screening [12]. Other than tumor biopsy, endoscopic ultrasound (EUS) is an important diagnostic procedure for a suspected rectal NEN, as recommended by the European Neuroendocrine Tumor Society (ENETS) [13]. Body computed tomography (CT) and magnetic resonance imaging (MRI) scans are required for the screening of distant metastases.

Surgical resection remains the major form of therapy for rectal NENs. In Japan, endoscopic resection procedures are recommended for tumors smaller than 1 cm, with no invasion of the muscularis propria and no lymph node metastases [14]. Chemotherapy is recommended for tumors showing distant metastases. Currently, two kinds of drugs are approved for rectal NENs in Japan: the mechanistic target of rapamycin (mTOR) inhibitor everolimus (brand name Afinitor), as well as the somatostatin analog lanreotide (brand name Somatulin) or octreotide (brand name Sandostatin) [14–16].

However, many questions remain unanswered in terms of the clinicopathology of rectal NENs. As described earlier, conclusive evidence is still lacking with regards to the various factors that predict the risk of distant metastasis, and therefore prognosis [17]. Further studies are also necessary to determine the pathological mechanisms affecting the growth, as well as local and distant metastasis, of primary rectal NENs. Last but not least, the indications for surveillance, as well as various forms of therapy (surgical resection, chemotherapy), remain unstandardized among countries, and are mostly based on experts’ opinions rather than case studies and large-scale prospective studies [18].

In this case study, we report the results from an autopsy conducted on a patient showing multiple distant metastases arising from a single G2 rectal NET. It is hoped that the findings could add to the current literature by providing answers to the key questions above.

## Case presentation

A 62-year-old Japanese woman with no prior medical or family history presented to the palliative care department of our hospital with pain and bloating of the upper abdominal region in December 2020.

**Fig. 1:**
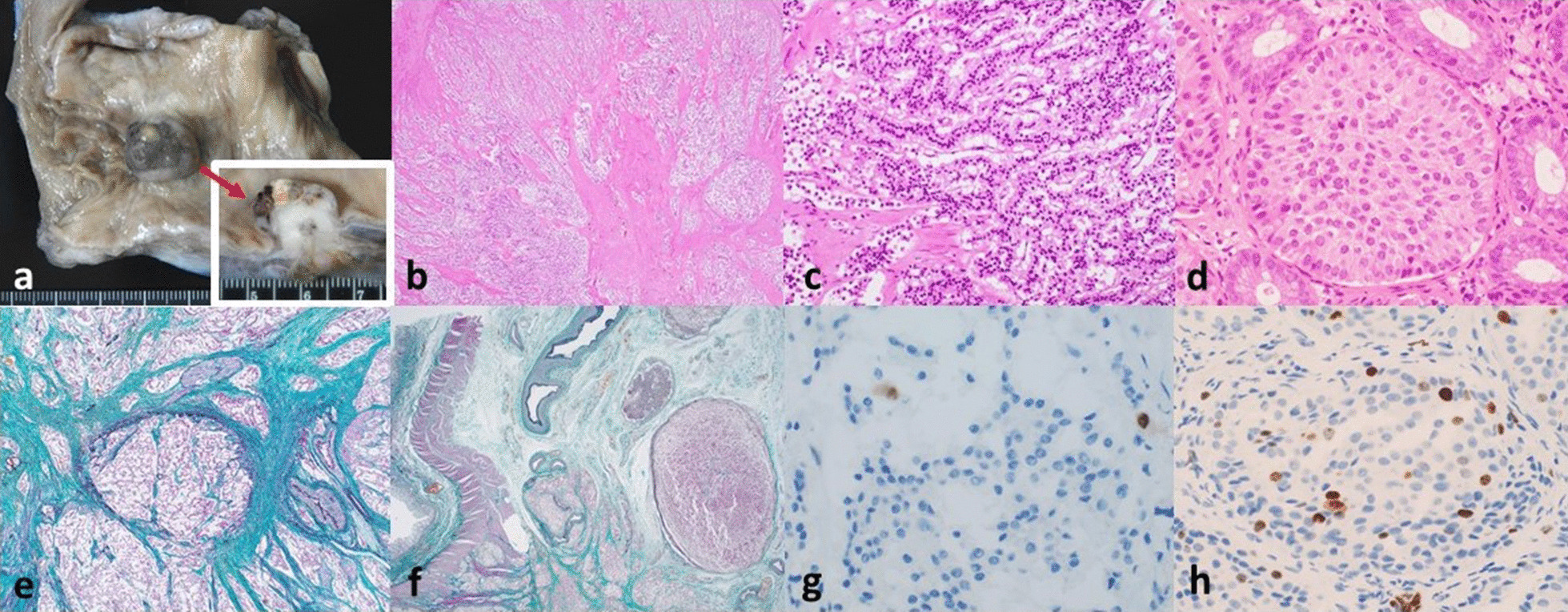
Rectum. **a** Gross appearance of rectum at autopsy stage, showing an 18 × 16 × 15 mm^3^ tumor with a black mucosal appearance at the Ra region. Dissection of the tumor revealed a white solid cross-sectional appearance (window, indicated by a red solid arrow). **b** Histologically, tumor cells were arranged in trabeculae with central scarring. **c** Higher magnification of histological appearance, showing cells arranged in trabeculae more clearly. **d** Histological appearance of rectal tumor biopsied in December 2018, for comparison purposes. Tumor cells were arranged in organoid and trabecular patterns. **e**, **f** Subserosa level of rectum at autopsy stage (Elastica–Masson staining). Multiple intravenous tumor embolisms were observed. **g** Ki-67 labeling index of rectal tumor at autopsy stage was 2%. **h** Ki-67 labeling index of rectal tumor biopsied in December 2018 was 4.9%

She was diagnosed with G2 rectal NET at an endoscopic clinic in December 2018. A biopsy of the rectum at that time revealed small regular cells with oval nuclei and eosinophilic granular cytoplasm, arranged in organoid and trabecular patterns (Fig. 1d). The cells were positive for synaptophysin, chromogranin A, as well as CD56 staining. Ki-67 labeling index was 4.9% (Fig. 1h). Serum neuron specific enolase (NSE), a highly specific tumor marker for NEN, was also high. These results supported the initial diagnosis.

An endoscopic mucosal resection (EMR) procedure was attempted at the same clinic the following month but without success. Multiple liver tumors, initially thought to be metastases, were discovered during a CT scan conducted the same month, and subsequently the patient was referred to the medical oncology department of a university hospital.

**Fig. 2:**
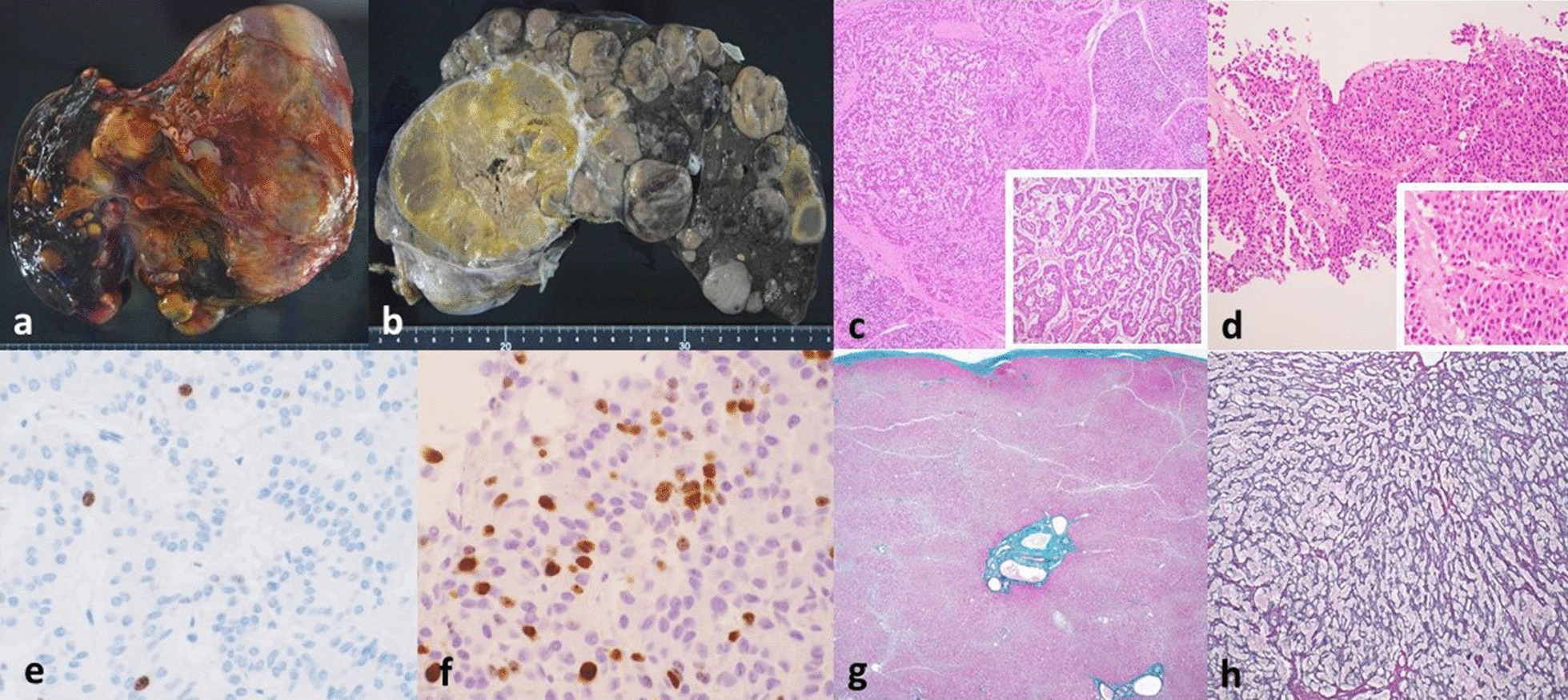
Liver. **a** Gross appearance of liver at autopsy stage. The liver weighed 5100 g. **b** Multiple milky-colored solid nodules with a capsular appearance, the largest of which measured 80 mm, could be observed on the cut-surface of the liver. **c** Histological appearance of liver tumors at autopsy stage. High magnification image (window) showed tumor cells arranged in trabeculae and rosettes, with a high level of necrosis. **d** Histological appearance of liver tumors biopsied in February 2019 for comparison. **e** Ki-67 labeling index of liver tumor at autopsy stage was 4%. **f** Ki-67 labeling index of liver tumor biopsied in February 2019 was 10.1%. **g** Elastica–Masson staining of normal liver tissue at autopsy stage, showing narrowing of hepatic cell cords. **h** Silver staining of normal liver tissue at autopsy stage, showing collapsing of hepatic cells, indicating chronic congestion

Additional liver biopsy conducted in February 2019 revealed cells with eosinophilic cytoplasm arranged in trabecular and rosette patterns (Fig. 2d). The cells were also positive for synaptophysin, chromogranin A, and CD56 staining. Ki-67 labeling index was 10.1% (Fig. 2f). These findings were consistent with the initial diagnosis that the liver tumors were metastases of the original rectal tumor.

From March 2019, the patient underwent several rounds of outpatient chemotherapy with the mTOR inhibitor everolimus. The results were favorable—a clinical evaluation of stable disease (SD) was made. From April 2020, the somatostatin analog lanreotide was added; however, shortly afterwards, the patient was hospitalized for unfavorable side effects. Postdischarge, everolimus therapy was reinitiated but was terminated 5 months later again due to unfavorable side effects. The patient and her family declined further chemotherapy, and was referred to our hospital for palliative care.

**Fig. 3:**
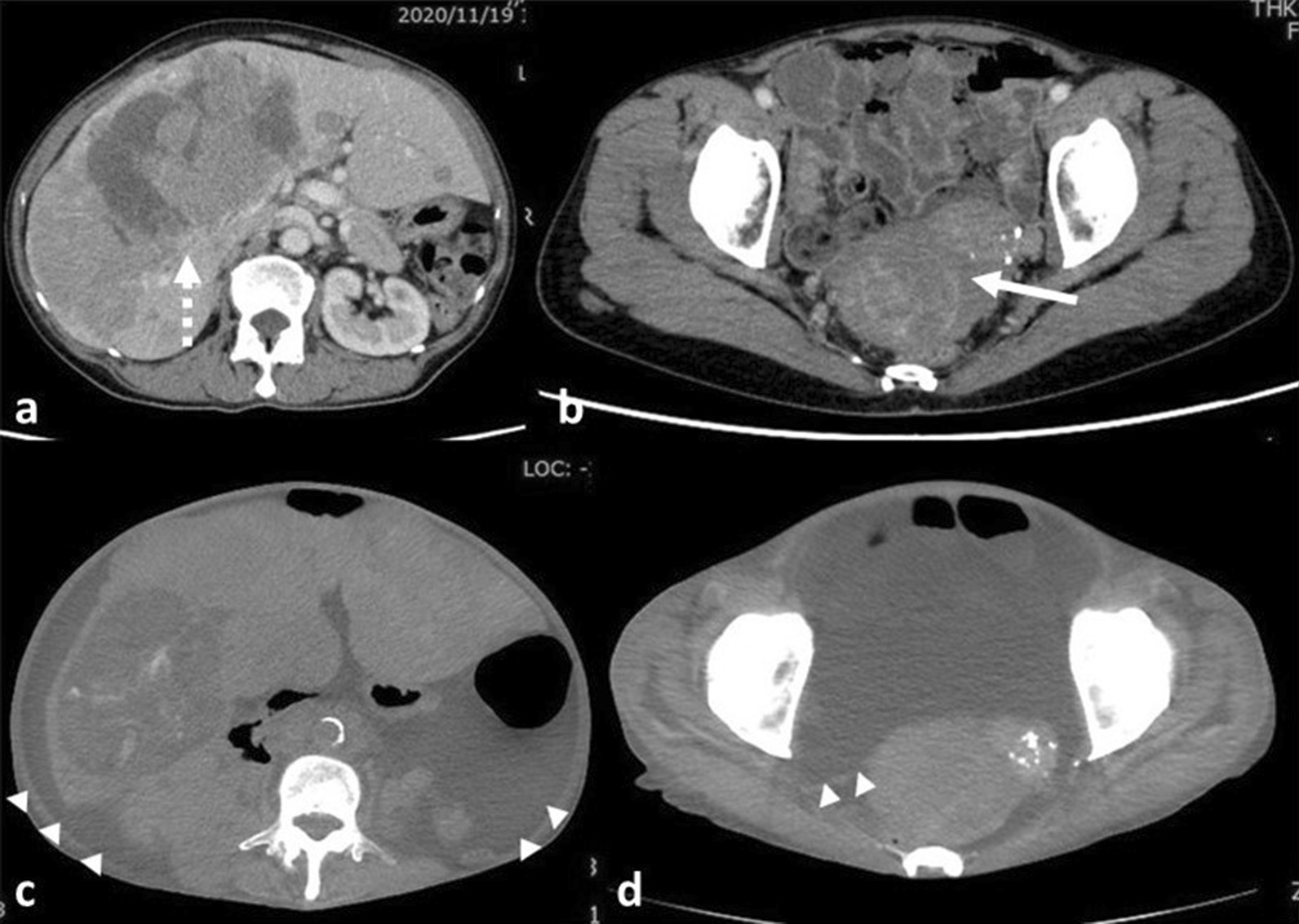
Computed tomography scans. **a**, **b** Enhanced body computed tomography scan taken in November 2020, showing a lesion 7 × 8 cm^2^ in size in the cavity of the lesser pelvis (solid arrow), as well as multiple liver metastases (dotted arrow). **c**, **d** Plain body computed tomography scan taken on 23 May 2022 (final hospitalization). The largest liver metastasis showed decreasing size, but ascites were present (arrowheads), and there were also findings of possible additional bone metastases (not shown)

An enhanced CT scan made in November 2020 (1 month prior to referral to our hospital) revealed a 7–8 cm lesion in the cavity of the lesser pelvis, which was deemed the site of the primary NET (Fig. 3a). However, the lesion could not be differentiated from a large uterine fibroid, which was also previously diagnosed, and therefore the actual size of the primary NET was inconclusive. Multiple tumors of varying enhancement levels, the largest of which measured 12 × 9 cm^2^, were found in the liver and thought to be metastases. No ascites was present (Fig. 3b).

Blood tests conducted during the patient’s early visit at our outpatient clinic revealed impaired liver function, possibly due to the liver metastases, as indicated by elevated aspartate transaminase (AST) and alanine transaminase (ALT) levels. Elevated lactate dehydrogenase(LDH) and creatine kinase (CK) levels suggested active proliferation and replacement of tumor cells. C-reactive protein (CRP) and white blood cell (WBC) levels were also high, which indicated inflammation due to the tumors (Table 1).

**Table 1:** Results of blood tests conducted on 21 January 2021 and 23 May 2022

	21/1/2021	23/5/2022/
Serum albumin (g/dl)	3.7	2.9^‡^
Total bilirubin (mg/dl)	1.0	1.8^†^
Aspartate transaminase (AST) (U/l)	432^†^	34
Alanine transaminase (ALT) (U/l)	67^†^	42
Lactate dehydrogenase (LDH) (U/l)	5318^†^	363^†^
γ-Glutamyltransferase (γ-GT) (U/l)	172^†^	308^†^
Alkaline phosphatase (ALP) (U/l)	346^†^	516^†^
Creatine kinase (CK) (U/l)	2230^†^	Unavailable
Blood urea nitrogen (BUN) (mg/dl)	21.3^†^	31.7^†^
Serum creatinine (Cr) (mg/dl)	0.63	0.68
Serum Na (mEq/l)	138	136
Serum K (mEq/l)	4.8	4.5
C-reactive protein (CRP) (mg/dl)	14.07^†^	0.31^†^
White blood cells (WBC) (× 10^3^/μl)	10.59^†^	5.94
Red blood cells (RBC) (× 10^6^/μl)	405	339^‡^
Hemoglobin (Hb) (g/dl)	11.0^‡^	10.6^‡^
Blood platelets (Plt) (× 10^4^/μl)	28.6	22.0

^†^Higher than normal range

^‡^Lower than normal range

As the patient had a performance status of 0 during her initial visit, best supportive care (BSC) was provided at the outpatient clinic, without hospitalization. Her chief complaints were pain and bloating of the upper abdominal region due to the liver metastases, and therefore pain control with fentanyl tape and oral oxycodone was initiated. She also complained of appetite loss as well as general malaise, and oral dexamethasone was also prescribed. The patient was followed at regular intervals at the outpatient clinic for the subsequent 17 months.

On 23 May 2022, the patient complained of increased abdominal pain, and was hospitalized. At the time, her performance status was 3. A plain CT scan revealed additional bone metastases, as well as a high level of peritoneal effusion. However, the liver metastases showed decreasing sizes (Fig. 3c, d). Even though she presented without fever, a diagnosis of suspected peritoneal carcinomatosis was made.

As the patient could not take oral opioids due to increased abdominal pain and bloating, she was switched to a course of continuous subcutaneous oxycodone infusion. Vital signs were stable during the next 2 days, and the patient reported limited pain. However, on the morning of 26 May 2022, the level of consciousness of the patient suddenly declined. While blood pressure levels and heart rates were stable, on physical examination the patient’s upper extremities were pale and cool, indicating unfavorable blood circulation levels. The patient’s family did not wish for further medical intervention, and at about 4 p.m. the same day, the patient died peacefully.

Several questions were raised regarding the clinical presentation of this patient. First, what was the pathological mechanism that underlies the multiple distant metastases from a single rectal NET in this patient? Second, was the diagnosis of suspected peritoneal carcinomatosis correct during the patient’s final hospitalization? Finally, the clinical progression during the last few days of this patient was relatively rapid, and differential diagnoses of hepatic failure from the multiple liver metastases, tumor embolism, as well as pharmacology-induced coma, could be made. Histologically, were there any of such findings in this patient?

To answer these questions, we requested an autopsy of the patient’s body. Both verbal and written consent were provided by the patient’s next-of-kin.

After legal pronunciation of death, the patient’s body was removed of any intubations and other medical devices, cleaned, and sent to the pathology department in the morning of 27 May 2022. The autopsy began roughly 18 hours after the patient’s death. The autopsy was conducted by two board-certified clinical pathologists and two assistants.

A gross examination of the patient’s body was first conducted. The patient’s body was then dissected and relevant organs, tissues, and body fluids were removed, weighed and measured, and preserved in formaldehyde solution for further inspection. According to the wishes of the patient’s family, no incisions were made on and around the facial region. All incision lines were then sewn and the body returned to the patient’s family for funeral purposes.

Photographs of the macroscopic appearances of the relevant organs and tissues were taken. The organs and tissues were further dissected and biopsy slides for microscopic examination were made.

### Outcomes

Unless otherwise stated, all biopsy slides were stained in hematoxylin–eosin solution.

#### Rectum

There was a single 18 × 16 × 15 mm^3^ tumor with a black mucosal appearance at the Ra region of the rectum, thought to be the primary tumor. On dissection, the tumor had a white solid appearance (Fig. 1a). Histologically, cells with deeply-stained chromatin were arranged in trabeculae with central scarring (Fig. 1b, c). Immunohistochemically, the cells were positive for chromogranin A, but were negative for synaptophysin. Cell mitosis was limited, and the Ki-67 labeling index was 2% (Fig. 1g).

At the subserosal layer of the rectum, tumor invasion and multiple intravenous tumor embolisms were found. Perineural invasion was also present (Fig. 1e, f). There was, however, no evidence of lymph vessel invasion.

#### Liver

The liver weighed 5100 g, nearly four times that of a normal human being. Multiple milky-colored solid nodules with a capsular appearance, the largest of which measured 80 mm, could be observed on the cut-surface (Fig. 2a, b). Microscopically, the nodules were composed of cells arranged in trabeculae and rosettes, similar to that of the primary tumor (Fig. 2c). The level of necrosis was high, and findings of fibrotic walls could also be seen. Ki-67 labeling index was 4% (Fig. 2e).

On the other hand, there was no histological evidence of portal vein inflammation or fibrosis in the nontumor regions of the liver; however, narrowing of the hepatic cell cords and collapsing of hepatic cells could be observed as chronic congestion (Fig. 2g, h). Cholestasis was unremarkable.

#### Pancreas

**Fig. 4:**
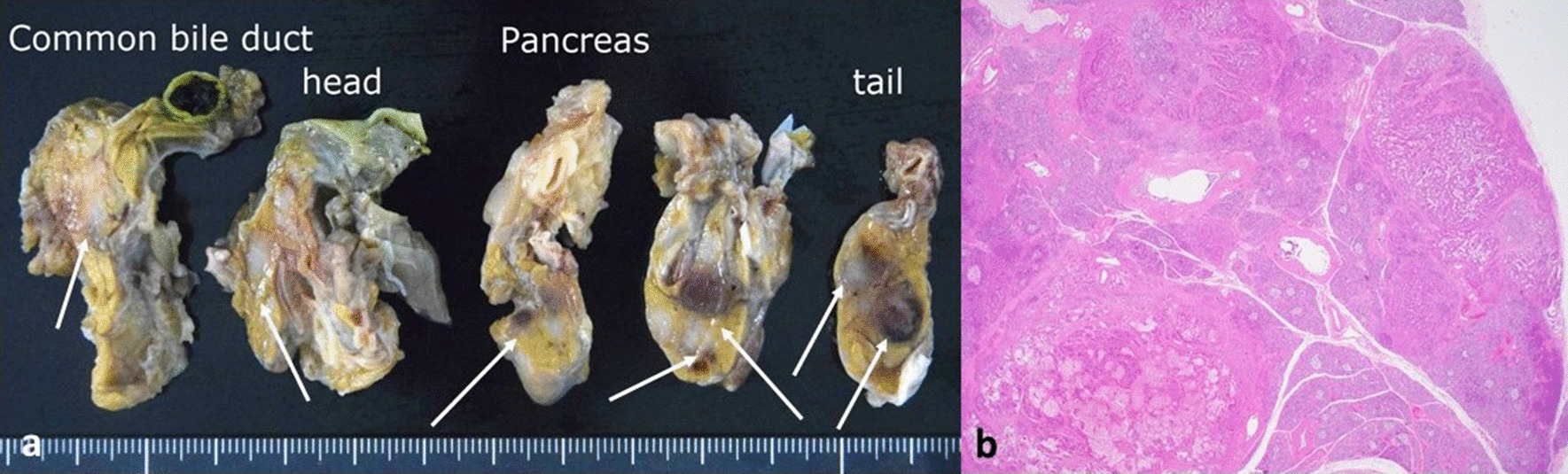
Pancreas. **a** Multiple metastatic nodules in the pancreatic parenchyma at autopsy stage (metastatic nodules shown by white solid arrows). **b** Histologically, tumor cells and hemorrhagic manifestations were present

The pancreas weighed 87 g. Multiple metastatic nodules could be observed, the largest of which measured 6.9 × 6.8 mm^2^ at the pancreatic tail, with hemorrhagic manifestations present microscopically (Fig. 4).

#### Other metastases

**Fig. 5:**
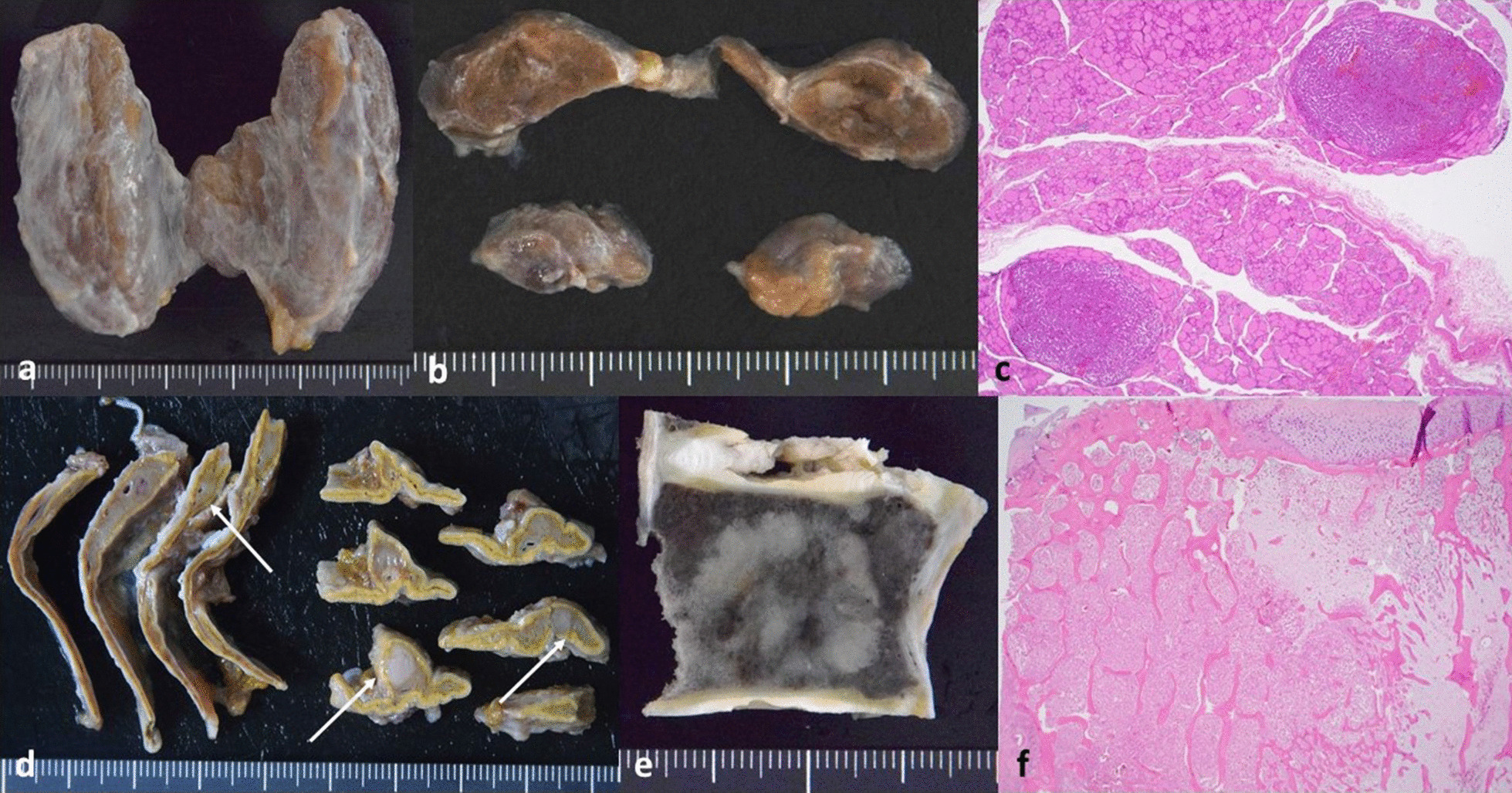
Other metastases. **a** Gross appearance of thyroid gland at autopsy stage. **b** Cut section of thyroid gland. Multiple metastatic nodules were present. **c** Microscopic findings confirmed the presence of tumor cells in the thyroid gland. **d** Cut sections of both adrenal glands. Metastatic nodules were present (white solid arrows). **e** Cut section of the vertebrae showing bone metastasis. **f** Histologically, tumor cells were present but the trabecular architecture was intact

Metastatic nodules could also be observed at the thyroid gland (Fig. 5a–c), both adrenal glands (Fig. 5d), as well as the vertebrae. The largest vertebrae metastasis measured 25 mm and had a solid appearance (Fig. 5e). Histologically, Ki-67 labeling index was around 2%, and there was relatively little trabecular fracture due to the bone metastases (Fig. 5f).

#### Other relevant findings

Multiple bilirubin stones, measuring 1–3 mm in diameter, were present in the gallbladder and common bile duct. Findings of chronic cholecystitis could also be made.

No tumor was demonstrated in the ascites.

**Fig. 6:**
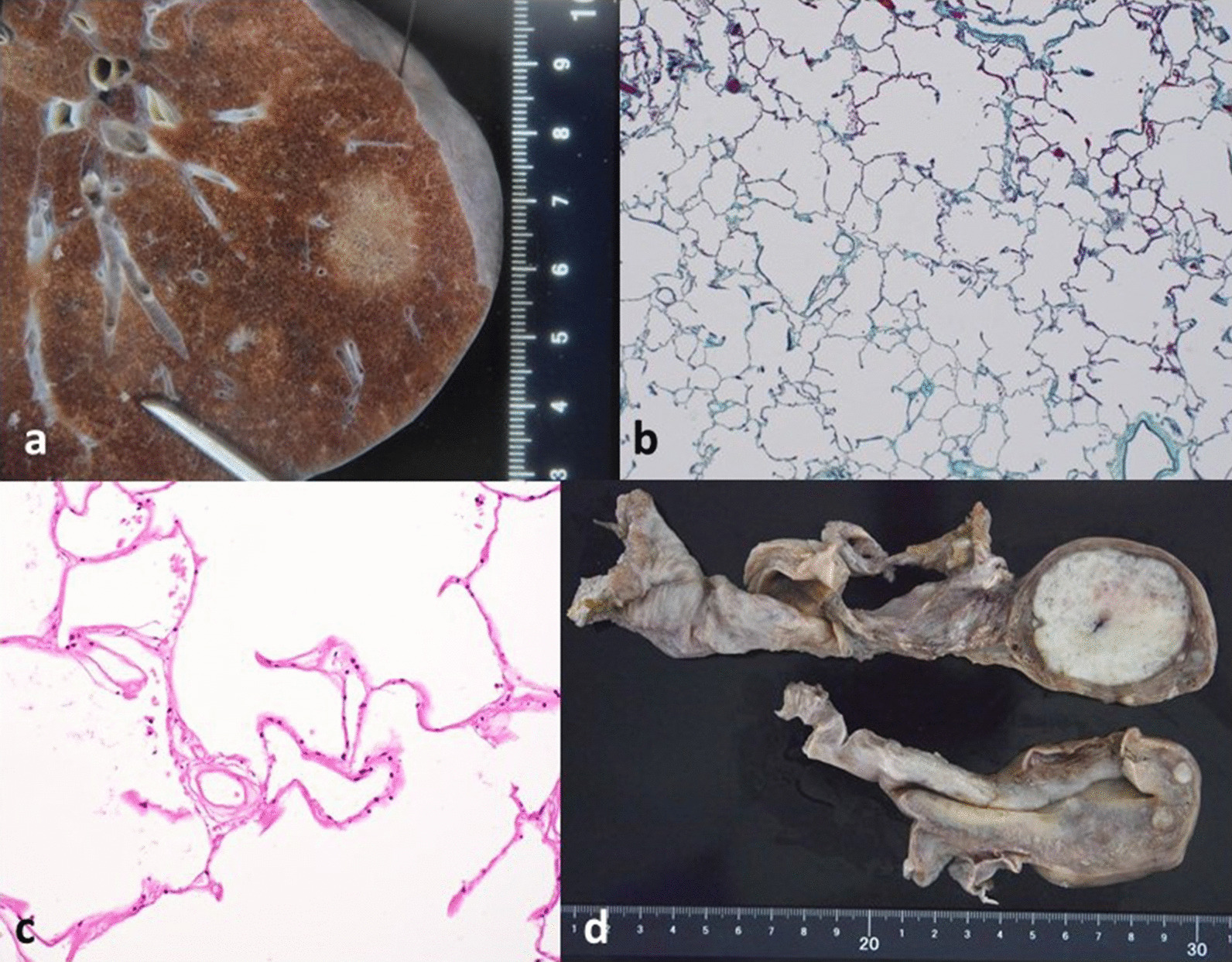
Other relevant findings. **a** Enlarged photograph of the S2 of the upper lobe of the left lung, showing a nodule with a ground-glass-like reticular appearance. **b** Microscopically, there were no tumor cells (Elastica–Masson staining). **c** Upon enlargement, disappearance of type II epithelial cells and reduction of elastic fibers were observed. **d** There were multiple uterine leiomyomas, the largest of which measured 50 mm

The S2 region of the left lung lobe corresponding to the B2aiβ segment of the bronchial branch consisted of a 17 × 14 × 14 mm^3^ nodule with a ground-glass-like reticular appearance, which was first thought to be a primary lepidic type lung adenocarcinoma (Fig. 6a). Histologically, disappearance of type II epithelial cells and reduction of elastic fibers were observed, suggesting tissue infarction rather than malignancy (Fig. 6b, c).

There were multiple uterine leiomyomas, the largest of which measured 50 mm, corresponding to the observations made radiologically (Fig. 6d).

## Discussion and conclusion

Here, we discuss the implications from the results. First, as we demonstrated in Figs. 1 and 2, histological findings suggest that the primary site is indeed the rectum. Curiously, while the observations made in December 2018 supported the diagnosis of G2 rectal NET, those measured upon death indicated that the rectal NET may be G1 at most, with a decrease in Ki-67 labeling index from 4.9 to 2%. Similarly, as in Figs. 3 and 4, the Ki-67 labeling index of the liver metastases also decreased, from 10.1% in February 2019 to 4% upon death.

There are two explanations for this. Arima *et al.* reported that prolonged fixation in formaldehyde may result in a time-dependent decrease in the Ki-67 labeling index of breast cancer specimens [19]. In our case, the immunohistochemical stain of liver tumor was made immediately after the autopsy, and therefore we considered that the impacts of formaldehyde preservation were minimal. Nevertheless, such a possibility could not be ruled out in its entirety.

More plausibly, however, the mitotic level and Ki-67 labeling index of the tumors may have been reduced in response to chemotherapy using mTOR inhibitors and somatostatin analogs. This is supported by Childs *et al.* and Vilar *et al.* [20, 21]. The relatively slow pace by which the disease progressed after chemotherapy in this case could also be a strong indicator that the level of malignancy of the primary tumor may have decreased. These implications reinforce the results of clinical studies, which concluded that mTOR inhibitors and somatostatin analogs are the most effective therapies for metastatic NENs of the digestive system [14–16].

The findings in the rectal subserosa suggest that the major pathological pathway by which the tumor cells metastasized in this case is transvenous and not translymphatic. Tumor embolisms could be observed in multiple venules, indicating that the level of venous invasion was high and contributed significantly to the eventual metastases. Histological findings support the conclusion that the tumors in the liver, pancreas, thyroid gland, adrenal glands, and vertebrae are distant metastases and not independent primary tumors.

This is an exceptionally rare case in which a single, small, low-grade rectal NET of low mitotic count and Ki-67 labeling index developed into multiple distant metastases. Toh *et al.*, Sasou *et al.* and Kim *et al.* made similar case reports, and while angiolymphatic invasion and multicentricity were identified as common risk factors for metastasis, the detailed mechanism was not explained [22–24].

In our case, we discovered that histologically, the liver metastases were large and accompanied by high levels of necrosis. The Ki-67 labeling index was almost twice that of the primary tumor. We hence deduced that the monoclonal tumor cells may have seeded at the liver via a venous pathway, and from there, mutated, gained multiclonality, and therefore, further metastatic potential and aggressiveness, and spread to other organs and tissues.

This hypothesis is reflected in other case studies—Jiang *et al.* reported in a meta-analysis that hepatoid adenocarcinoma cells of the stomach may develop multiclonal architecture associated with liver metastasis [25]. Hoadley *et al.* also found that mutation and multiclonal seeding may be the major mechanism affecting the risk of metastasis in basal-like breast cancer [26]. Unfortunately, we did not explore the molecular genetic relationships between the primary tumor and the metastatic cells, and this remains a topic for future discussion.

The results of the cytological examination of the ascites, as well as other histological findings, indicate that the major cause of death was tumor cell death-related cachexia and not peritoneal carcinomatosis. While there was elevation of liver enzyme levels during the patient’s final hospitalization, it was perhaps more due to biliary tract obstruction from stones rather than hepatic failure or drug-induced liver damage.

One of the major strengths of this case study is that being a postmortem investigation, it provided valuable *in vivo* evidence for the fundamental clinicopathology of rectal NETs and the major pathways in which they may metastasize to other tissues and organs. As stated above, multiple distant metastases from a small, single rectal NET, such as this case, are rarely reported, and so we hope that the insights from this case study will lead to further research into better diagnostic and therapeutic methods in the future.

This study is not, however, without its limitations. First, being a singular case study, it must be emphasized that the findings may not be applicable to all diagnosed cases of rectal NENs. Second, the autopsy was conducted nearly 18 hours after the patient was legally pronounced dead, and certain parts of relevant tissues and organs could not be examined due to autolysis.

In conclusion, we reported a rare and interesting case of multiple liver, pancreas, thyroid gland, adrenal glands, and vertebrae metastases arising from a single, small G2 NET of the rectum. Postmortem examination of the patient’s body suggested that vascular invasion may have been the major pathological mechanism by which tumor cells metastasized to the liver, during which the tumor cells may have gained multiclonality, and further metastatic potential. While findings of tumor embolism are present, the main cause of death is concluded to be tumor cell death-related cachexia. We hope that the results of this study could shed light on the clinicopathology of rectal NENs, and pave the way for further research into more effective diagnostic and therapeutic methods for this rare disease in the future.

## Data Availability

The data and images generated or analyzed during the current study are available from the corresponding author on reasonable request.

## Abbreviations

CT: Computed tomography
NEC: Neuroendocrine carcinoma
NEN: Neuroendocrine neoplasm
NET: Neuroendocrine tumor
NSE: Neuron specific enolase
SD: Stable disease
